# Short-term and Long-term Rates of Postacute Sequelae of SARS-CoV-2 Infection: A Systematic Review

**DOI:** 10.1001/jamanetworkopen.2021.28568

**Published:** 2021-10-13

**Authors:** Destin Groff, Ashley Sun, Anna E. Ssentongo, Djibril M. Ba, Nicholas Parsons, Govinda R. Poudel, Alain Lekoubou, John S. Oh, Jessica E. Ericson, Paddy Ssentongo, Vernon M. Chinchilli

**Affiliations:** 1 Department of Surgery, Penn State College of Medicine and Milton S. Hershey Medical Center, Hershey, Pennsylvania; 2 Department of Public Health Sciences, Penn State College of Medicine and Milton S. Hershey Medical Center, Hershey, Pennsylvania; 3 Cognitive Neuroscience Unit, School of Psychology, Deakin University, Melbourne, Victoria, Australia; 4 Mary Mackillop Institute for Health Research, Department of Health Sciences, Australian Catholic University, Melbourne, Victoria, Australia; 5 Department of Neurology, Penn State College of Medicine and Milton S. Hershey Medical Center, Hershey, Pennsylvania; 6 Division of Infectious Disease, Department of Pediatrics, Penn State College of Medicine and Milton S. Hershey Medical Center, Hershey, Pennsylvania; 7 Center for Neural Engineering, Department of Engineering, Science and Mechanics, The Pennsylvania State University, State College

## Abstract

**Importance:**

Short-term and long-term persistent postacute sequelae of COVID-19 (PASC) have not been systematically evaluated. The incidence and evolution of PASC are dependent on time from infection, organ systems and tissue affected, vaccination status, variant of the virus, and geographic region.

**Objective:**

To estimate organ system–specific frequency and evolution of PASC.

**Evidence Review:**

PubMed (MEDLINE), Scopus, the World Health Organization Global Literature on Coronavirus Disease, and CoronaCentral databases were searched from December 2019 through March 2021. A total of 2100 studies were identified from databases and through cited references. Studies providing data on PASC in children and adults were included. The Preferred Reporting Items for Systematic Reviews and Meta-analyses (PRISMA) guidelines for abstracting data were followed and performed independently by 2 reviewers. Quality was assessed using the Newcastle-Ottawa Scale for cohort studies. The main outcome was frequency of PASC diagnosed by (1) laboratory investigation, (2) radiologic pathology, and (3) clinical signs and symptoms. PASC were classified by organ system, ie, neurologic; cardiovascular; respiratory; digestive; dermatologic; and ear, nose, and throat as well as mental health, constitutional symptoms, and functional mobility.

**Findings:**

From a total of 2100 studies identified, 57 studies with 250 351 survivors of COVID-19 met inclusion criteria. The mean (SD) age of survivors was 54.4 (8.9) years, 140 196 (56%) were male, and 197 777 (79%) were hospitalized during acute COVID-19. High-income countries contributed 45 studies (79%). The median (IQR) proportion of COVID-19 survivors experiencing at least 1 PASC was 54.0% (45.0%-69.0%; 13 studies) at 1 month (short-term), 55.0% (34.8%-65.5%; 38 studies) at 2 to 5 months (intermediate-term), and 54.0% (31.0%-67.0%; 9 studies) at 6 or more months (long-term). Most prevalent pulmonary sequelae, neurologic disorders, mental health disorders, functional mobility impairments, and general and constitutional symptoms were chest imaging abnormality (median [IQR], 62.2% [45.8%-76.5%]), difficulty concentrating (median [IQR], 23.8% [20.4%-25.9%]), generalized anxiety disorder (median [IQR], 29.6% [14.0%-44.0%]), general functional impairments (median [IQR], 44.0% [23.4%-62.6%]), and fatigue or muscle weakness (median [IQR], 37.5% [25.4%-54.5%]), respectively. Other frequently reported symptoms included cardiac, dermatologic, digestive, and ear, nose, and throat disorders.

**Conclusions and Relevance:**

In this systematic review, more than half of COVID-19 survivors experienced PASC 6 months after recovery. The most common PASC involved functional mobility impairments, pulmonary abnormalities, and mental health disorders. These long-term PASC effects occur on a scale that could overwhelm existing health care capacity, particularly in low- and middle-income countries.

## Key Points

**Question:**

What are the short-term and long-term postacute sequelae of COVID-19 (PASC) infection?

**Findings:**

In this systematic review of 57 studies comprising more than 250 000 survivors of COVID-19, most sequelae included mental health, pulmonary, and neurologic disorders, which were prevalent longer than 6 months after SARS-CoV-2 exposure.

**Meaning:**

These findings suggest that long-term PASC must be factored into existing health care systems, especially in low- and middle-income countries.

## Teaser

This systematic review estimates organ system–specific frequency and evolution of postacute sequelae of COVID-19 infection.

## Introduction

The global COVID-19 pandemic that began in late 2019 has caused more than 187 million infections and 4 million deaths as of July 10, 2021.^1^ Survivors experience long-lasting medical, psychological, and economic consequences, further increasing the disability-adjusted life years lost.^2^ Despite current vaccination efforts,^3^ the health consequences of COVID-19 remain urgent, with long-term multi-organ system impacts that are yet to be elucidated. With a variety of clinical presentations and degrees of severity in patients,^4^ there is a dire need to better understand the lasting and emergent effects of COVID-19.

Frequently reported residual effects from SARS-CoV-2 virus include fatigue, dyspnea, chest pain, persistent loss of taste and/or smell, cognitive changes, arthralgias, and decreased quality of life. Many of these symptoms may result from widespread neuropathological events occurring in major white matter bundle tracts, cortical gray matter, and subcortical gray matter.^5^ In a study conducted in the United States by Chopra et al,^6^ 33% of patients had persistent symptoms at a 60-day follow-up after COVID-19 hospitalization. Similar trends have been observed in Europe.^7^ Furthermore, persistent symptoms (>6 weeks) have been reported in 19% of fully vaccinated individuals.^8^ However, as the pandemic emerged in 2019, most studies have been limited in the duration of observation, and there has yet to be a consolidation of these trends to portray an overarching evolution of these symptoms from short-term to long-term sequelae following COVID-19 infection.

To our knowledge, short-term and long-term sequelae of COVID-19 have not been systematically evaluated. In this paper, we synthesized the existing literature to estimate the overall and organ system–specific frequency of postacute sequelae of COVID-19 (PASC). We sorted studies into groups that focused on (1) postacute symptoms at 1-month after acute COVID-19 (short term), (2) persisting and new clinical manifestations between 2 and 5 months after infection (intermediate term), and (3) clinical manifestations that were present at least 6 months after COVID-19 (long term). These categorizations were based on literature reports proposing a framework that COVID-19 infection progresses from an acute infection lasting approximately 2 weeks into a postacute hyperinflammatory illness lasting approximately 4 weeks, until ultimately entering late sequelae.^9,10^ As we better understand the disease burden of PASC in COVID-19 survivors, we can develop precise treatment plans to improve clinical care in patients with COVID-19 who are at greatest risk of PASC and establish integrated, evidence-based clinical management for those affected.

## Methods

### Information Source and Search Strategy

The present study has been prospectively registered at PROSPERO (CRD42021239708) and followed the Preferred Reporting Items for Systematic Reviews and Meta-analyses (PRISMA) reporting guideline.^11^ Databases were searched from December 2019 through March 2021, including PubMed (MEDLINE), Scopus, the World Health Organization Global Literature on Coronavirus Disease, and CoronaCentral. We manually searched the reference lists of included studies and other relevant documents to find additional studies. There were no limitations on country of publication or language. Non–English language articles were translated using the language translation services at the Penn State University Library. Predefined search terms included multiple combinations of the following: (*COVID-19* OR *coronavirus* OR *SARS-CoV-2* OR *2019-nCoV* OR *SARS nCoV2*) AND (*post-acute sequelae of SARS-COV-2* OR *long COVID-19* OR *post-COVID-19 syndrome*). Studies obtained from the search were transferred into EndNote version 9.3.2 (Clarivate), and duplicates were removed.

### Eligibility and Inclusion Criteria

Studies were selected according to the following criteria: participants, adults and children with a previous COVID-19 infection; exposure, COVID-19; condition or outcome of interest, frequency of PASC; study design and context, randomized clinical trials, prospective and retrospective cohort studies, case series with at least 10 patients, and case-control studies. Inclusion criteria included the following: previous COVID-19 diagnosis and reported PASC frequencies.

### Data Extraction

Two investigators (D.G. and A.S.) screened titles and abstracts of all identified articles for eligibility. Full-text articles were screened from eligible studies. Disagreements were resolved by discussion with a third investigator (P.S.). The following information was extracted by 2 investigators (D.G. and A.S.) independently: year of publication, country and time frame of the study, sample size of survivors of COVID-19, number of participants with PASC, mean (SD) or median (IQR) age, percentage male, percentage hospitalized, outcome of interest, time zero (ie, from diagnosis of COVID-19 or hospital discharge), and measurement methods for outcome of interest.

### Study Quality Assessment

Two reviewers (D.G. and A.S.) independently assessed the quality of the included studies. The Newcastle-Ottawa Scale (NOS) was used for the quality assessment of the included studies.^12^ Based on the NOS criteria, we assigned a maximum of 4 stars for selection, 2 stars for comparability, and 3 stars for exposure and outcome assessment. Studies with fewer than 5 stars were considered low quality; 5 to 7 stars, moderate quality; and more than 7 stars, high quality.

### Definition of Short-term, Intermediate-term, and Long-term PASC

The primary outcome was the frequency of PASC, which was defined as the presence of at least 1 abnormality diagnosed by (1) laboratory investigation, (2) radiologic pathology, or (3) clinical signs and symptoms that was present at least 1 month after COVID-19 diagnosis or after discharge from the hospital. We defined short-term PASC as 1 month; intermediate-term, 2 to 5 months; and long-term, as 6 or more months after COVID-19 diagnosis or hospital discharge.

### Statistical Analysis

A narrative approach was used to describe the number of studies, proportion male, proportion hospitalized, median or mean age (by study), whether the study was conducted in low- and middle-income countries (median gross national income, ≤$12 535) or high-income countries (median gross national income, ≥$12 536). We did not conduct a meta-analysis due to high heterogeneity in the outcome of interest. We summarized PASC rates descriptively, reporting medians and IQRs. PASC frequencies were summarized as short term, intermediate term, or long term and by organ system. R package ggplot2 was used to display the boxplots.^13^ All statistical analyses were performed with R software version 3.6.2 (R Project for Statistical Computing).

## Results

### Identified Studies

As shown in eFigure 1 in the Supplement, we identified a total of 2100 studies. After excluding the duplicates and studies that did not meet inclusion criteria after screening the title, abstract, or main text, a total of 57 studies were included, with 250 351 survivors of COVID-19 who were assessed for PASC at 30 days after acute COVID-19 infection and beyond. The mean (SD) age of survivors was 54.4 (8.9) years, 140 196 (56%) were male, and 197 777 (79%) were hospitalized during acute COVID-19. High-income countries contributed 45 studies (79%). Study-specific details are provided in the Table.^6,7,14,15,16,17,18,19,20,21,22,23,24,25,26,27,28,29,30,31,32,33,34,35,36,37,38,39,40,41,42,43,44,45,46,47,48,49,50,51,52,53,54,55,56,57,58,59,60,61,62,63,64,65,66,67,68^

**Table.** Study Specific Details

Source	Country	Study type	Baseline	Timeframe, mo	Quality score	Outcome measurements	Male, %	Age, mean (SD), y	Hospitalized, %	PASC, No.	Sample size, No.
Carvalho-Schneider et al,^14^ 2021	France	Prospective cohort	Diagnosis with confirmed laboratory result	1	5	mMRC dyspnea scale (dyspnea), self-reported symptoms scaled on 10-point analog scale (chest pain, anosmia, and ageusia)	43	49 (15)	29	103	150
Glück et al,^15^ 2021	Germany	Prospective cohort	Diagnosis, with confirmed laboratory result	1	7	Serum laboratory tests, self-reported symptoms (fever, nausea, diarrhea, loss of smell or taste, fatigue, dyspnea, headache, cough, runny nose, sore throat, myalgia), enzyme-linked immunosorbent assay	38	Median, 40	NA	67	119
Pellaud et al,^16^ 2020	Switzerland	Retrospective cohort	Diagnosis with confirmed laboratory result and hospital admission	1	5	Self-reported over telephone interview	61	Median (IQR), 70 (60-80)	100	73	196
Akter et al,^17^ 2020	Bangladesh	Cross-sectional	Diagnosis with confirmed laboratory result	1	5	Medical records; self-report over telephone interview	76	NA	100	675	734
Panda et al,^18^ 2020	India	Prospective cohort	Diagnosis with confirmed laboratory result and hospital admission	1	6	Self-reported over telephone interview	71	35 (13)	100	210	225
Huang et al,^19^ 2020	China	Retrospective cohort	Hospital discharge	1	8	Medical records, lung radiography (chest abnormalities), 6MWT (functional status), spirometry (lung function)	46	46 (14)	100	31	57
Jacobs et al,^20^ 2020	US	Prospective cohort	Hospital discharge	1	5	Self-reported symptoms, PROMIS Scale version1.2; Global Health and Item Bank version 1.0; Dyspnea Functional Limitations Short Form 10a	61.5	Median (IQR), 57 (48-68)	100	82	183
Poncet-Megemont et al,^21^ 2020	France	Retrospective cohort	Diagnosis (laboratory result or positive CT)	1	5	Self-reported symptoms from telephone interview	13	49 (15)	45	20	139
Weerahandi et al,^22^ 2021	United States	Prospective cohort	Hospital discharge	1	5	Self-report	57	57	100	113	152
Daher et al,^23^ 2020	Germany	Prospective cohort	Hospital discharge	1.5	6	Body plethysmography, serum laboratory tests, lung diffusion capacity, ABG, 6MWT, echocardiography, laboratory tests, quality of life (PHQ-9, GAD-7, SGRQ, and EQ-5D-5L)	67	64 (3)	100	15	33
de Graaf et al,^24^ 2021	Netherlands	Prospective cohort	Hospital discharge	1.5	7	Echocardiography, ECG monitoring, pulmonary function testing, GAD-7, PHQ-9, PCL-5, CFQ-25, IQ-CODE-N, PCFS	63	60.8 (13)	42	55	81
Tomasoni et al,^25^ 2021	Italy	Cross-sectional	Hospital discharge	1.5	5	Self-reported symptoms, HADS (mental status), MMSE (cognitive disorders)	73	Median (IQR), 55 (43-65)	100	55	105
Chiesa-Estomba et al,^26^ 2020	Spain	Prospective cohort	Diagnosis	1.5	7	Short Questionnaire of Olfactory Disorders–Negative Statements and self-reported ENT, olfactory, and gustatory dysfunction	36	41 (13)	100	384	751
Chopra et al,^6^ 2021	US	Prospective cohort	Hospital discharge	2	6	Medical records	52	Median (IQR), 62 (50-72)	100	159	488
Mendez et al,^27^ 2021	Spain	Prospective cohort	Hospital discharge	2	7	Quality of Life (SF-12), verbal memory (SCIP), verbal fluency (ANT), working memory (WAIS-III), anxiety (GAD-7), depression (PHQ-2), PTSD (DTS)	58.7	Median (IQR), 57 (49-67)	100	79	179
Huang et al,^28^ 2021	United States	Retrospective cohort	Diagnosis (with confirmed laboratory result)	2	7	Medical records	28	NA	NA	380	1407
Smet et al,^29^ 2021	Belgium	Retrospective cohort	Diagnosis	2	6	Lung radiography (chest abnormalities), spirometry (lung function), laboratory data (lactate dehydrogenase, troponin, D-dimer)	62	55 (13)	NA	137	220
Sonnweber et al,^30^ 2020	Austria	Prospective cohort	Diagnosis	2	5	Self-reported symptoms, 6MWT (functional mobility), blood test	60	58 (14)	80	32	109
Vaira et al,^31^ 2020	Italy	Prospective cohort	Diagnosis	2	5	Olfactory and gustatory psychophysical tests	49.3	51.2 (8.8)	23	8	138
Carvalho-Schneider et al,^14^ 2021	France	Prospective cohort	Diagnosis with confirmed laboratory result	2	5	mMRC Dyspnea Scale (dyspnea), self-reported symptoms scaled on 10-point analog scale (chest pain, anosmia, and ageusia)	44	49 (15)	28	86	130
Puntmann et al,^32^ 2020	Germany	Prospective cohort	Diagnosis with confirmed laboratory result	2	8	MRI (cardiac activity), laboratory data (cardiac activity), self-reported (other outcomes)	53	49 (14)	33	78	100
Carfi et al,^7^ 2021	Italy	Prospective cohort	Hospital discharge	2	5	EQ-VAS (QOL); self-reported symptoms in patient survey	63	57 (15)	100	125	143
Rosales-Castillo et al,^33^ 2021	Spain	Retrospective cohort	Diagnosis with confirmed laboratory result	2	5	Self-reported symptoms	56	60 (15)	100	74	118
Halpin et al,^34^ 2021	UK	Prospective cohort	Hospital discharge	2	5	EQ-5D-5L (QOL); telephone interview screening tool (other outcomes)	54	Median (range), 71 (20-93)	100	64	100
Islam et al,^35^ 2021	UK	Prospective cohort	Diagnosis within 7 d of hospital admission	2	6	Self-reported symptoms via survey	52	Median (IQR), 66 (52-80)	100	114	403
D’Cruz et al,^36^ 2021	UK	Prospective cohort	Diagnosis at hospital admission	2	6	mMRC Dyspnea Scale (dyspnea); PHQ-9 (depression); TSQ (trauma); GAD-7 (anxiety); 6-CIT (cognitive impairment); CT scan (organ function); 4MGS (gait speed); 1-min sit-to-stand test (mobility)	62	59 (14)	100	106	119
Mandal et al,^37^ 2021	UK	Prospective cohort	Diagnosis upon hospital admission	2	6	Lung radiography (chest abnormalities); blood sample (laboratory assessments); PHQ-2 (depression); self-reported symptoms	62	60 (16)	100	276	384
Raman et al,^38^ 2021	UK	Prospective cohort	Hospital discharge	2.5	7	Radiographic imaging, spirometry, 6MWT (functional mobility), CPET (cardiopulmonary fitness), QOL, self-reported health assessment	58.6	55.4 (13.2)	100	54	58
Shah et al,^39^ 2021	Canada	Prospective cohort	Diagnosis with confirmed laboratory result	3	8	Pulmonary function test (lung function); 6MWT (mobility); CT scan (organ function); UCSD SOBQ (dyspnea)	68	Median (IQR), 67 (54-74)	100	53	60
Wong et al,^40^ 2020	Canada	Prospective cohort	Diagnosis with confirmed laboratory result	3	8	EQ-5D-5L (QOL); UCSD Frailty Index (frailty); UCSD SOBQ (shortness of breath); PSQI (sleep quality); PHQ-9 (depression), self-reported symptoms via survey	64	62 (16)	100	59	78
Taquet et al,^41^ 2021	US	Retrospective cohort	Diagnosis	3	8	Medical records	44	46 (20)	20	78 005	236 379
Tabatabaei et al,^42^ 2020	Iran	Retrospective cohort	Diagnosis with chest CT	3	6	Medical records, laboratory data (SpO_2_, white blood cell, C-reactive protein, lactate dehydrogenase, leukocytosis), CT imaging	62	50 (13)	81	22	52
Glück et al,^15^ 2021	Germany	Prospective cohort	Diagnosis	3	7	Serum laboratory tests, self-reported symptoms (fever, nausea, diarrhea, loss of smell or taste, fatigue, dyspnea, headache, cough, runny nose, sore throat, myalgia), enzyme-linked immunosorbent assay	38	Median, 40	NA	29	119
Townsend et al,^43^ 2020	Ireland	Prospective cohort	Acute illness recovery	3	7	CFQ-11 (fatigue), laboratory results (white blood cell, C-reactive protein, lactate dehydrogenase, interleukin 6, soluble interleukin-2 receptor)	46	50 (15)	55	67	128
Janiri et al,^44^ 2021	Italy	Prospective cohort	Acute illness recovery	3	7	Clinician-Administered PTSD Scale, self-reported COVID-19 characteristics	56	55 (15)	81	306	381
van den Borst et al,^45^ 2020	Netherlands	Prospective cohort	Hospital discharge	3	6	Pulse-oximetry and spirometry (pulmonary functioning); mMRC Dyspnea Scale (dyspnea); CT scan and radiography (chest function); CFS (frailty); HADS (anxiety and depression); TICS and CFQ (cognitive function); PCL-5 and IES-R (PTSD); SF-36 (QOL); blood sample (laboratory assessments)	60	59 (14)	100	89	124
Lerum et al,^46^ 2021	Norway	Prospective cohort	Hospital admission	3	5	Self-report: mMRC Dyspnea Scale, QOL (EQ-5D-5L), chest CT scan, pulmonary function tests (spirometry)	54	Median (IQR), 59 (49-72)	NA	37	103
Sibila et al,^47^ 2021	Spain	Prospective cohort	Hospital admission	3	4	Pulmonary function tests (spirometry and DLCO)	57	56 (16)	100	109	172
Arnold et al,^48^ 2021	UK	Prospective cohort	Hospital admission	3	6	Chest radiograph, pulmonary function tests (spirometry), exercise testing, serum laboratory tests, QOL (SF-36), WEMWBS	62	NA	100	81	110
Zhao et al,^49^ 2020	China	Retrospective cohort	Diagnosis or symptom onset	3	6	Medical records, chest CT, pulmonary function tests, serum laboratory tests	58	NA	NA	35	55
Weng et al,^50^ 2021	China	Prospective cohort	Hospital admission	3	3	Self-reported symptoms (fever, cough, dyspnea, gastrointestinal), medical records	56	NA	100	52	117
Xiong et al,^51^ 2021	China	Prospective cohort	Hospital discharge	3	8	Medical records, self-report symptoms (general, respiratory, cardiovascular, psychological, and specifics)	46	Median (IQR), 52 (41-62)	100	267	538
Liang et al,^52^ 2020	China	Prospective cohort	Hospital discharge	3	8	Self-reported symptoms, serum laboratory tests, pulmonary function tests, high-resolution CT imaging	28	41.3 (13.8)	100	45	76
Qu et al,^53^ 2021	China	Prospective cohort	Hospital discharge	3	5	Self-reported symptoms from phone interview, medical records for laboratory results, HRQoL (QOL)	50	Median (IQR), 47.5 (37-57)	100	311	540
Sonnweber et al,^54^ 2021	Austria	Prospective cohort	Hospital discharge	3	5	Self-reported, mMRC score (dyspnea), spirometry (lung function), lung and chest radiography, laboratory tests	55	57 (14)	75	59	145
Ugurlu et al,^55^ 2021	Turkey	Prospective cohort	Diagnosis, ie, laboratory result	3	5	Self-reported symptoms, B-SIT (smell abnormalities)	45	41 (14)	100	42	104
Peluso et al,^56^ 2021	US	Prospective cohort	Diagnosis or symptom onset	4	5	Somatic symptoms (PHQ), QOL (EuroQol), mental health (GAD-7, PHQ-8, PCL-5)	56	Median (IQR), 48 (38-55)	37	65	119
Garrigues et al,^57^ 2020	UK	Prospective cohort	Hospital admission	4	6	mMRC Dyspnea Scale; QOL (EQ-5D-5L); health state (EQ-VAS)	75	63 (16)	100	66	120
Bellan et al,^58^ 2021	Italy	Prospective cohort	Hospital discharge	4	8	Pulmonary function tests, physical performance (SPPB), PTSD (IES-R)	60	Median (IQR), 61 (50-71)	31	238	767
Moreno-Perez et al,^59^ 2021	Spain	Prospective cohort	Diagnosis or symptom onset	4	8	QOL (EQ-VAS), chest radiographs, serum laboratory tests, pulmonary function tests	53	Median (IQR), 56 (53-72)	66	141	277
Guler et al,^60^ 2021	Switzerland	Prospective cohort	Acute illness recovery	4	6	Medical records, pulmonary function tests (spirometry, DLCO, respiratory strength), chest CT	59	NA	NA	37	113
Dennis et al,^61^ 2021	UK	Prospective cohort	Diagnosis or symptom onset	5	8	Self-report, serum laboratory tests, MRI, QOL (EQ-5D-5L)	30	44 (11)	18	199	201
Logue et al,^62^ 2021	US	Prospective cohort	Diagnosis or symptom onset	6	5	Self-reported symptoms	43	48 (15)	NA	55	177
Rauch et al,^63^ 2021	Germany	Prospective cohort	Diagnosis or symptom onset	6	5	Self-reported symptoms	32	NA	9	85	127
Trunfio et al,^64^ 2021	Italy	Retrospective cross-sectional	Diagnosis or symptom onset	6	8	Self-reported symptoms	56	Median (IQR), 56 (43-69)	64	41	200
Walle-Hansen et al,^65^ 2021	Norway	Prospective cohort	Hospital admission	6	5	QOL (EQ-5D-5L), VAS, cognitive capacity (MoCA), functional capacity (SPPB)	57	74	100	57	106
Huang et al,^66^ 2021	China	Ambidirectional cohort	Diagnosis or symptom onset	6	8	Dyspnea (mMRC), QOL, anxiety, and depression (EQ-5D-5L and EQ-VAS), serum laboratory tests, CT scans, mobility (6MWT)	52	Median (range), 57 (0-65)	NA	1265	1655
Han et al,^67^ 2021	China	Prospective cohort	Diagnosis or symptom onset	6	8	Medical records, chest CT, pulmonary function tests (spirometry, DLCO)	70	54 (12)	62	40	114
Taboada et al,^68^ 2021	Spain	Prospective cohort	Hospital discharge	6	5	HRQoL (QOL), functional status, self-reported symptoms	59	65.5 (10.4)	100	61	91
Peluso et al,^56^ 2021	US	Prospective cohort	Diagnosis or symptom onset	8	5	Somatic symptoms (PHQ), QOL (EuroQol), mental health (GAD-7, PHQ-8, PCL-5)	56	Median (IQR), 48 (38-55)	69	48	64
Glück et al,^15^ 2021	Germany	Prospective cohort	After COVID-19 diagnosis	8	7	Serum laboratory work, self-reported symptoms (fever, nausea, diarrhea, loss of smell or taste, fatigue, dyspnea, headache, cough, runny nose, sore throat, myalgia), enzyme-linked immunosorbent assay	38	Median, 40	0	35	119

Abbreviations: 4MGS, 4-meter gait speed; 6-CIT, 6-item Cognitive Impairment Test; 6MWT, 6-minute walk test; ABG, arterial blood gas; ANT, Animal Naming Test; B-SIT, Brief Smell Identification Test; CFS, Clinical Frailty Scale; CFQ, Cognitive Failures Questionnaire–25; CPET, cardiopulmonary exercise test; CT, computed tomography; DLCO, diffusing capacity for carbon monoxide; DTS, Davidson Trauma Scale; ENT, ear, nose, and throat; ECG, electrocardiogram; EQ-5D-5L, EuroQol 5-level 5-dimension; EQ-VAS, EuroQol visual analog scale; GAD-7, General Anxiety Disorder–7; HADS, Hospital Anxiety and Depression Scale; HRQoL, health-related quality of life; IES-R, Impact of Events Scale; IQ-CODE-N, Informant Questionnaire on Cognitive Decline in the Elderly–Netherlands; mMRC, modified Medical Research Council; MMSE, Mini-Mental State Examination; MoCA, Montreal Cognitive Assessment; MRI, magnetic resonance imaging; NA, not available; PASC, post-acute sequelae of SARS-CoV-2 infection; PCL-5, PTSD Checklist of *DSM-5*; PCFS, Post–COVID-19 Functional Status; PHQ-2, Patient Health Questionnaire; PROMIS, Patient-Reported Outcomes Measurement Information System; PSQI, Pittsburgh Sleep Quality Index; PTSD, posttraumatic stress disorder; QOL, quality of life; SCIP, Screen for Cognitive Impairment in Psychiatry; SF, Short Form; SGRQ, St George Respiratory Questionnaire; SpO_2_, peripheral capillary oxygen saturation; SOBQ, Shortness of Breath Questionnaire; SPPB, Short Physical Performance Battery; TICS, Telephone Interview for Cognitive Status; TSQ, Trauma Screening Questionnaire; UCSD, University of California, San Diego; WAIS-III, Wechsler Adult Intelligence Scale, third edition; WEMWBS, Warwick-Edinburgh Mental Well-being Scales.

### Frequency of PASC

**Figure 1.**
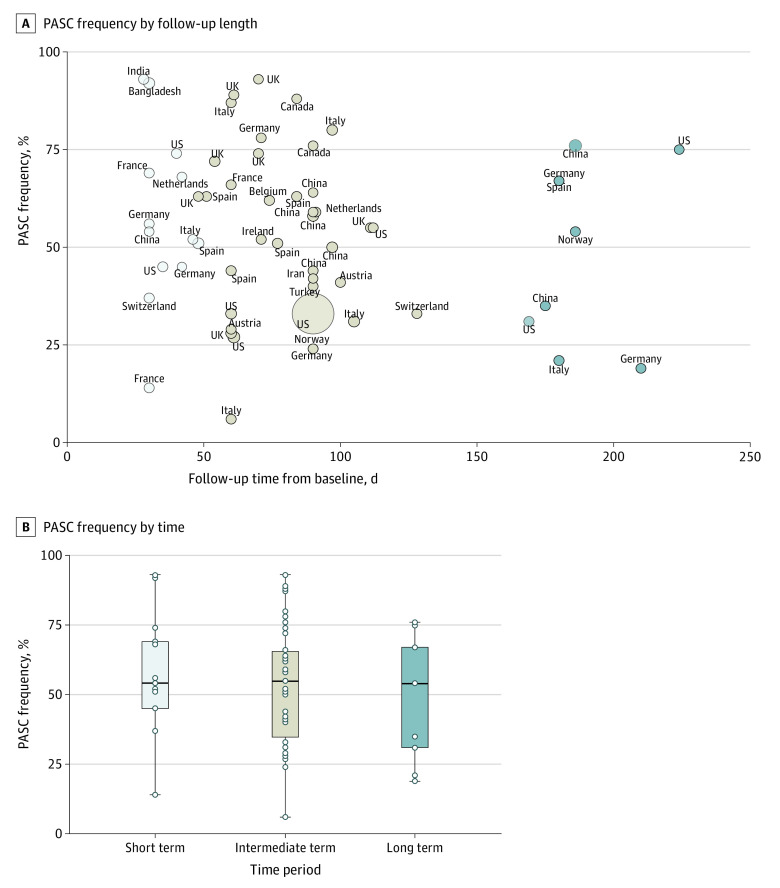
Studies Included Studying Postacute Sequelae of COVID-19 (PASC) A, Scatterplot representing each study’s PASC frequency (%) plotted according to length of follow-up from baseline (in days), represented by a circle proportional to the study’s sample size and annotated according to country. B, Box plot representing the frequency of PASC reported by follow-up period. The horizontal bar in each box plot is the median value for the outcome of interest. The edges of the box represent the first and third quartiles. The width of the box is the IQR. The whiskers extend to the smallest and largest observations within 1.5 times the IQR of the quartiles. The circles represent point estimates for each study included in the analysis. Circles extending beyond the whiskers are outliers.

Displayed in Figure 1A is the distribution of studies by country and follow-up time from baseline. PASC frequencies were stratified and reported by 1 month (short-term),^14,15,16,17,18,19,20,21,22,23,24,25,26^ 2 to 5 months (intermediate-term),^7,15,19,27,28,29,30,31,32,33,34,35,36,37,38,39,40,41,42,43,44,45,46,47,49,50,51,52,53,54,55,56,57,58,59,60,61,66,67^ and 6 months (long-term)^15,56,62,63,64,65,66,67^ from COVID-19 diagnosis or hospital discharge (Figure 1B). The median (IQR) proportion of COVID-19 survivors experiencing at least 1 PASC at 1 month was 54.0% (45.0%-69.0%; 13 studies); at 2-5 months, 55.0% (34.8%-65.5%; 38 studies); and at 6 or more months, 54.0% (31.0%- 67.0%; 9 studies). When stratified by World Bank income groups, median (IQR) PASC frequency was 54.6% (33.0%-68.3%; 45 studies) in high-income countries and 56.0% (43.5%-67.0%; 12 studies) for low- and middle-income countries (eFigure 2A in the Supplement). PASC rates were similar in studies with higher (≥60%) and lower (<60%) percentages of hospitalized patients (eFigure 2B in the Supplement). In addition, when stratified by study methodological score, the proportion of PASC were similar (eFigure 2C in the Supplement).

### Rates of Clinical Manifestations of PASC

A total of 38 clinical manifestations were assessed. We collapsed these clinical manifestations into categories of (1) organ systems, ie, neurologic, mental health, respiratory, cardiovascular, digestive, dermatologic, and ear, nose, and throat; (2) constitutional symptoms; and (3) functional mobility.

### Neurologic Symptoms

**Figure 2.**
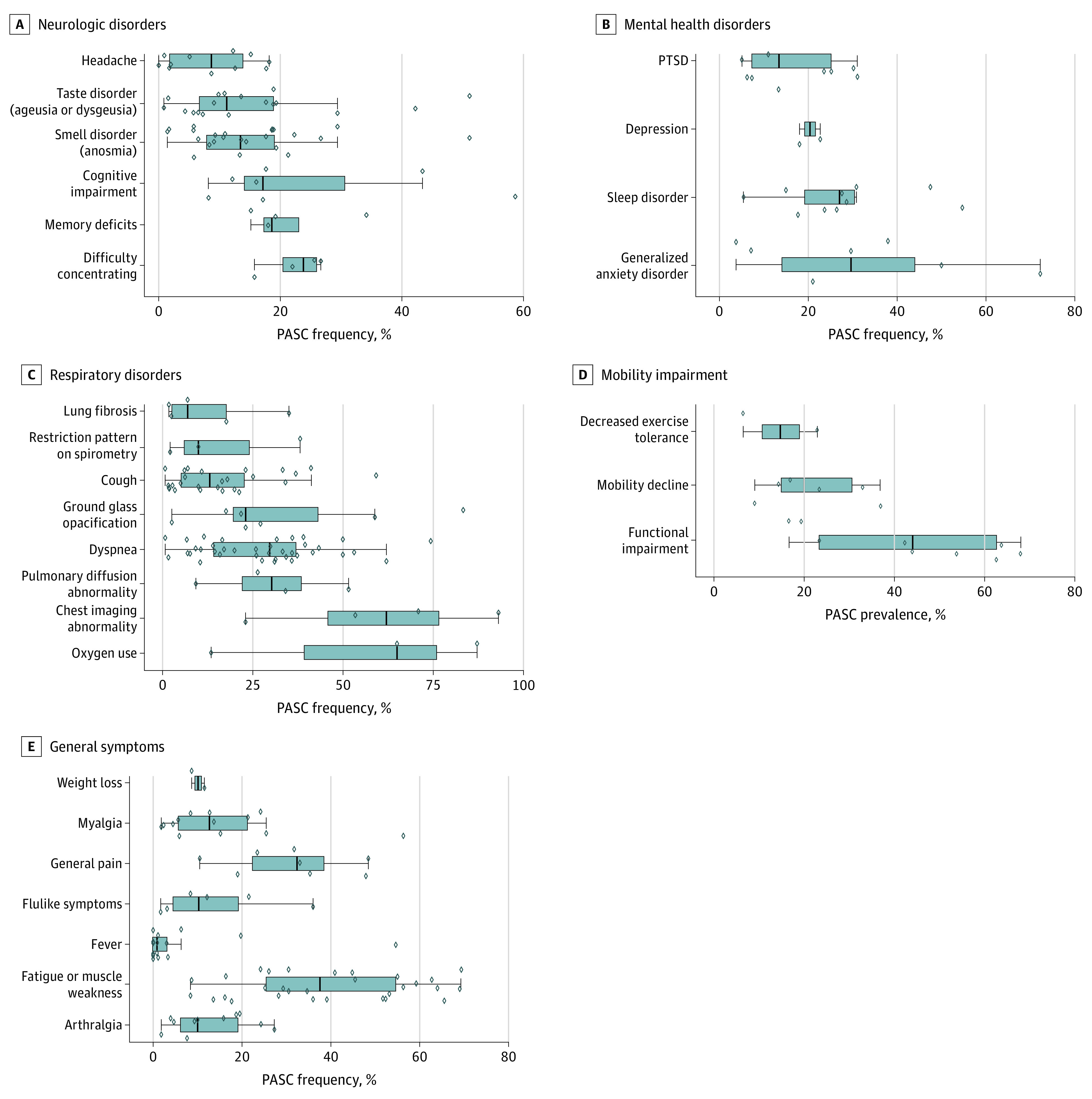
Neurologic, Mental Health, Respiratory, Mobility, and General Postacute Sequelae of COVID-19 (PASC) Symptoms The vertical bar in each box plot is the median value for the outcome of interest. The edges of the box represent the first and third quartiles. The width of the box is the IQR. The whiskers extend to the smallest and largest observations within 1.5 times the IQR of the quartiles. The diamonds represent point estimates for each study included in the analysis. Diamonds extending beyond the whiskers are outliers. PTSD indicates posttraumatic stress disorder.

Various neurologic symptoms were reported (Figure 2A). These included headaches, memory deficits, difficulty concentrating, and cognitive impairment. Even though anosmia (loss of smell) and ageusia or dysgeusia (loss or distortion of taste) are often reported as part of ear nose and throat system, we chose to include them in the neurologic symptoms because they are a consequence of the effect of the virus on the cranial nerve 1 (olfactory nerve) for smell and cranial nerves VII (facial), IX (glossopharyngeal nerve), and X (vagal nerve) for taste. The most common neurocognitive symptoms were difficulty concentrating (4 studies; median [IQR], 23.8% [20.4%-25.9%]), memory deficits (4 studies; median [IQR], 18.6% [17.3%-22.9%]), cognitive impairment (7 studies; median [IQR], 17.1% [14.1%-30.5%]). Dysgeusia and anosmia were reported in 11% (18 studies; median [IQR], 11.2% [6.7%-18.9%]) and 13% (24 studies; median [IQR], 13.4% [7.9%-19.0%]) of the survivors, respectively. Overall, headache symptoms were reported in 8% (11 studies; median [IQR], 8.7% [1.9%-13.9%]) of COVID-19 survivors. However, disparities existed in headache symptoms by study, ranging from 0% in Bellan and colleagues^58^ to 18% in Zhao et al.^49^

### Mental Health Disorders

A variety of standardized instruments were used to assess mental health. These included the Patient Health Questionnaire (PHQ) 2 to screen for depression, the PHQ 9 to evaluate major depressive disorder, the General Anxiety Disorder 7 to assess generalized anxiety disorder, the Hospital Anxiety and Depression Scale to measure symptoms of anxiety and depression, and the PTSD Checklist of *DSM-5* and the Impact of Events Scale to assess the presence and severity of posttraumatic stress disorder symptoms. The Pittsburgh Sleep Quality Index questionnaire was used to assess sleep quality and disturbances (Table). Depression or anxiety were reported in 9 studies, and the rates were consistent (Figure 2B). Approximately 1 in 3 COVID-19 survivors was diagnosed with generalized anxiety disorders (7 studies; median [IQR], 29.6% [14.0%-44.0%]), 1 in 4 with sleep disorders (10 studies; median [IQR], 27.0% [19.2%-30.3%]), 1 in 5 with depression (2 studies; median [IQR], 20.4% [19.2%-21.5%]), and 1 in 8 with posttraumatic stress disorder (9 studies; median [IQR], 13.3% [7.3%-25.1%]).

### Pulmonary Abnormalities

Pulmonary manifestations of PASC were assessed with pulmonary function tests (such as spirometry, diffusing capacity for carbon monoxide, and respiratory strength) and imaging modalities including chest radiograph, computed tomography scans, and magnetic resonance imaging. Dyspnea was mainly assessed with the Modified Medical Research Council Dyspnea Scale. Dyspnea was reported in 38 studies (median [IQR], 29.7%; [14.2%-37.0%]), and cough was reported in 26 studies (median [IQR], 13.1% [5.3%-22.6%]). Increased oxygen requirement was reported in nearly two-thirds of COVID-19 survivors (3 studies; median [IQR], 65.0% [39.3%-76.1%]). Other frequently reported sequelae included pulmonary diffusion abnormalities (4 studies; median [IQR], 30.3% [22.1%-38.5%]), ground glass opacification (7 studies; median [IQR], 23.1% [19.7%-43.0%]), restrictive patterns on spirometry (3 studies; median [IQR], 10.0% [6.1%-24.1%]), and lung fibrosis (5 studies; median [IQR], 7.0% [2.5%-17.7%]) (Figure 2C). Overall, chest imaging abnormalities were present in a median (IQR) of 62.2% (45.8%-76.5%) of survivors (4 studies).

### Functional Mobility Impairment

Three functional mobility impairments were assessed in this systematic review. They were impairment in general functioning (9 studies; median [IQR], 44.0% [23.4%-62.6%]), mobility decline (6 studies; median [IQR], 20.2% [14.9%-30.6%]), and reduced exercise tolerance (2 studies; median [IQR], 14.7% [10.6%-18.8%]) (Figure 2D).

### General and Constitutional Symptoms

Due to their subjective nature and self-reportage of symptoms (Table), general well-being and constitutional symptoms varied widely between studies. In this category, we noted 7 persisting symptoms among survivors of COVID-19 (Figure 2E). These included fatigue or muscle weakness, joint pain, muscle pain, flu-like symptoms, fever, general pain, and weight loss. Most commonly reported symptoms were joint pain (11 studies; median [IQR], 10.0% [6.1%-19.0%]), fatigue or muscle weakness (30 studies; median [IQR], 37.5% [25.4%-54.5%]), and flu-like symptoms (6 studies; median [IQR], 10.3% [4.5%-19.2%]). General pain (8 studies; median [IQR], 32.4% [22.3%-38.4%]), persistent fever (16 studies; median [IQR], 0.9% [0%-3.1%]), and muscle pain (13 studies; median [IQR], 12.7% [5.6%-21.3%]) were also frequently reported among survivors. Fever rates decreased as a function of time: by 60 days of follow-up, persistent fever rates reduced from 3% to 0% in studies by Carvalho-Schneider and colleagues.^14^ Except for Glück et al^15^ at a 1-month follow-up, the reported fever rates were less than 20%. The high fever rates reported in Glück et al^15^ can potentially be explained by unusually high anti–SARS-CoV-2 immunoglobulin G levels in their patient population of frontline health care workers, which was significantly associated with the severity of disease as reported by the authors. Fever rates for the subsequent follow-ups at 3, 5, and more than 6 months after diagnosis were all at 0% in the Glück study.^15^ Carvalho-Schneider et al^14^ reported a slight increase in unintentional weight loss (defined as a loss of more than or equal to 5% of body weight at baseline) from 9% to 12% at day 30 to day 60 of follow-up, respectively.

### Cardiovascular Disorders

**Figure 3.**
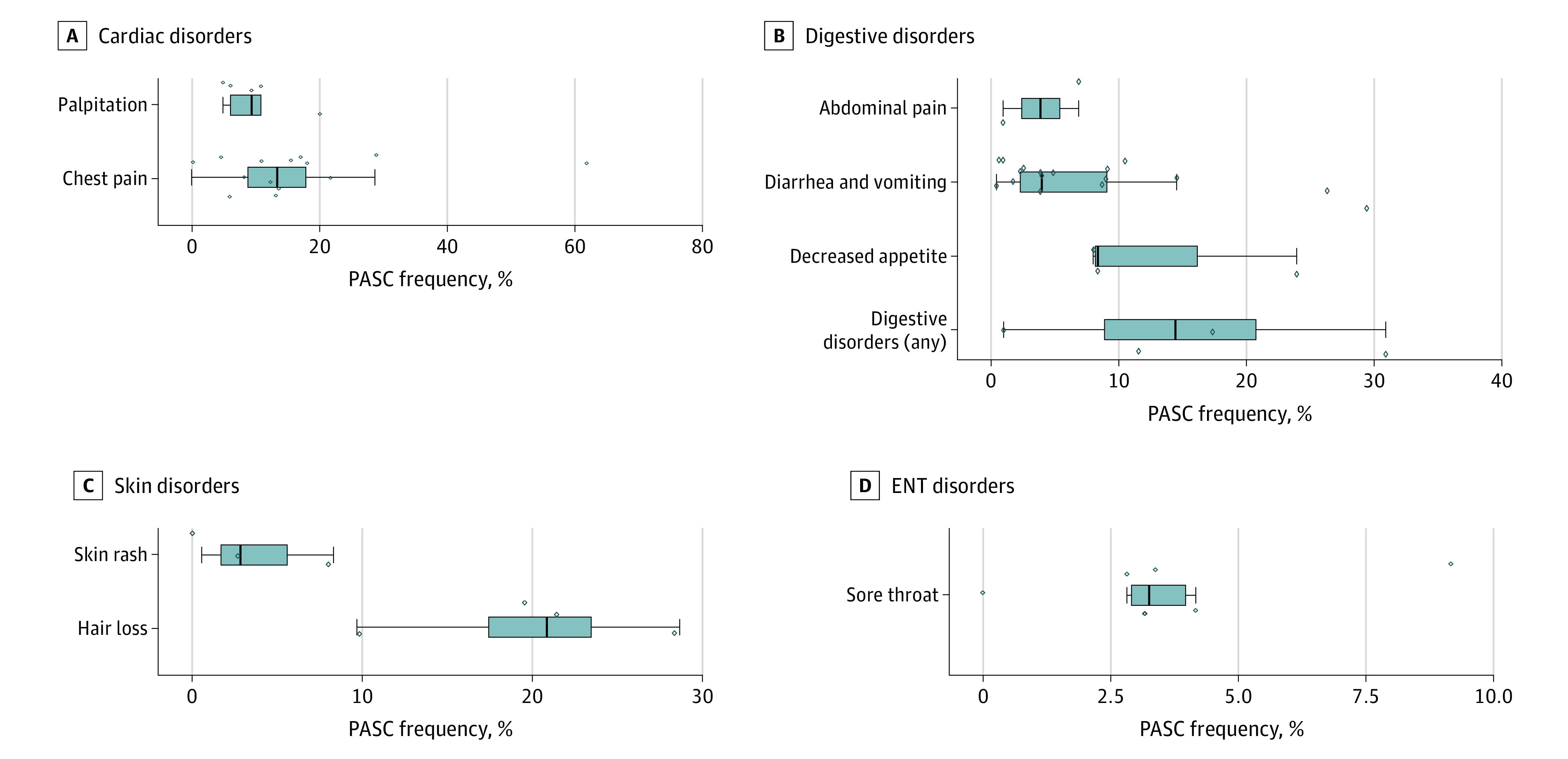
Cardiac, Digestive, Skin, and Ear, Nose, and Throat (ENT) Postacute Sequelae of COVID-19 (PASC) Symptoms The vertical bar in each box plot is the median value for the outcome of interest. The edges of the box represent the first and third quartiles. The width of the box is the IQR. The whiskers extend to the smallest and largest observations within 1.5 times the IQR of the quartiles. The diamonds represent point estimates for each study included in the analysis. Diamonds extending beyond the whiskers are outliers.

Chest pain and palpitations were common cardiovascular manifestations in survivors of COVID-19 (Figure 3A). The median (IQR) frequency of chest pain and palpitation were 13.3% (8.8%-17.8%; 14 studies) and 9.3% (6.0%-10.8%; 5 studies), respectively. Other reported diagnoses, such as myocardial infarction and heart failure, were not as frequently reported in the literature.

### Gastrointestinal, Dermatologic, and Ear, Nose, and Throat Disorders

The overall rate of gastrointestinal disorders was 6% and included abdominal pain, decreased appetite, diarrhea, and vomiting (Figure 3B). Hair loss (4 studies; median [IQR], 20.8% [17.4%-23.4%]) and skin rash (3 studies; median [IQR], 2.8% [1.7%-5.6%]) constituted dermatologic disorders (Figure 3C). Finally, sore throat was a concern among 3% of COVID-19 survivors (6 studies; median [IQR], 3.3%, [2.9%-4.0%]) (Figure 3D).

## Discussion

In this systematic review, we evaluated the temporal progression of clinical abnormalities experienced by patients who recovered from an infection with SARS-CoV-2, starting with a mean of 30 days post–acute illness and beyond. The results suggest that rates of PASC are indeed common; 5 of 10 survivors of COVID-19 developed a broad array of pulmonary and extrapulmonary clinical manifestations, including nervous system and neurocognitive disorders, mental health disorders, cardiovascular disorders, gastrointestinal disorders, skin disorders, and signs and symptoms related to poor general well-being, including malaise, fatigue, musculoskeletal pain, and reduced quality of life. Short- and long-term rates of PASC were similar, highlighting the potential for pathological sequelae long after exposure to the SARS-CoV-2 virus.

The mechanisms underpinning the postacute and chronic manifestations of COVID-19 are not entirely understood. Nevertheless, these mechanisms can be grouped into the direct effect of the viral infection and the indirect effect on mental health due to posttraumatic stress, social isolation, and economic factors, such as loss of employment.^69,70^ Direct viral effects can be explained by several hypotheses, including persistent viremia due to immune fatigue and paresis,^71^ relapse or reinfection,^72^ hyperinflammatory immune response, cytokine- and hypoxia-induced injury,^73^ and autoimmunity^74^ as well as neurotropism using a transsynaptic spread mechanism,^5^ resulting in hypoxic- or hemorrhagic-driven neuronal apoptosis.^75^ Herein, widespread acute injury to cortical/subcortical and white matter fiber bundles may affect brain function and impede distal brain connectivity, respectively, manifesting in common symptoms, such as those identified in this review. These symptoms may include headache (ie, encephalopathy), cognitive deficits (ie, widespread neuropathological events), and smell and taste disorders (ie, acute injury to olfactory bulb).

At the forefront of clinical care for acute COVID-19 are multiple guidelines, recommendations, and best practices that have been disseminated and prioritized for prevention and management. However, no clear guidelines are currently available for postinfectious care or recovery, and there is a notable dearth of information on and strategies about how to assess and manage patients following their acute COVID-19 episode. This is in part due to a high degree of between-study heterogeneity in defining PASC. Indeed, this heterogeneity was evident the present study. We noted varying definitions of time zero, which included symptom onset, COVID-19 diagnosis, hospital admission, or hospital discharge. Furthermore, variations in the specific outcomes of interest and the outcome measurement tools existed, hindering us from pooling the data in a formal meta-analytic model. SARS-CoV-2 variant types and breakthrough infectivity rates among fully vaccinated individuals will likely modify the manifestations and incidence of PASC further.^8^

Our results indicate that clinical management of PASC will require a whole-patient perspective, including management tools like virtual rehabilitation platforms and chronic care for post–acute COVID-19 symptoms in conjunction with the management of preexisting^76,77^ or new comorbidities.^78^ One-stop multidisciplinary clinics are therefore recommended to avoid multiple referrals to different specialists and encourage comprehensive care. Based on our work and the recent systematic reviews by Nasserie and colleagues,^79^ these specialists should include respiratory physicians, cardiologists, neurologists, general physicians (from primary care or rehabilitation medicine), neuropsychologists or neuropsychiatrists, physiotherapists, occupational therapists, speech and language therapists, and dieticians.^80^

The clinical and public health implications of our findings are 2-fold. In addition to the life lost from acute COVID-19 illness, many individuals experience disability due to PASC, greatly exacerbating the disease burden.^81^ Such a burden is more than enough to overwhelm existing health care system capacities, particularly in resource-constrained settings. Second, predictive models of postacute and chronic COVID-19 sequalae using clinical and laboratory data obtained during the acute phase of COVID-19 are critically needed to inform effective strategies to mitigate or prevent PASC.

### Limitations

This study has limitations. First, there is no consensus on the definition of postacute COVID-19. PASC currently has many definitions, including (1) the presence of symptoms beyond 3 weeks from the initial onset of symptoms^78^; (2) symptoms that develop during or following an infection consistent with COVID-19, continue for more than 4 weeks, and are not explained by an alternative diagnosis^80^; and (3) signs and symptoms at 12 weeks after infection and beyond. This led to considerable heterogeneity in PASC definitions among the articles synthesized in this systematic review. Therefore, it was difficult to precisely compare the percentages of patients with abnormalities on follow-up visits between studies and to obtain a standardized understanding of patients’ long-term symptoms from COVID-19. Second, we were not able to stratify the risk of PASC by severity of initial illness (for example, community-based vs hospitalized vs required care in an intensive care unit vs required invasive life-sustaining measures) or by preexisting comorbidities, patient age, or other factors that may affect an individual patient’s risk of PASC. Third, the lack of standard reporting also created differences in how PASC sequelae were analyzed. Fourth, many studies investigated the prevalence of specific outcomes instead of reporting all symptoms present at various points post-COVID-19 infection. This limits the ability for a comprehensive, generalizable analysis of the long-term effects of COVID-19. Fifth, many studies included in this analysis were obtained from manual searching through references. This might suggest a need for improved database search terms for subsequent studies.

## Conclusions

These findings suggest that PASC is a multisystem disease, with high prevalence in both short-term and long-term periods. These long-term PASC effects occurred on a scale sufficient to overwhelm existing health care capacity, particularly in resource-constrained settings. Moving forward, clinicians may consider having a low threshold for PASC and must work toward a holistic clinical framework to deal with direct and indirect effects of SARS-CoV-2 sequalae.

## Notes

Supplement. **eFigure 1. **Flow Diagram for Systematic Review of PASC **eFigure 2. **PASC Frequencies Stratified by National Income Level, Proportion Hospitalized, and Study Methodological Quality
