# Supplementary material for: Short-term and Long-term Rates of Postacute Sequelae of SARS-CoV-2 Infection: A Systematic Review
Source: JAMA Netw Open. 2021 Oct 13;4(10):e2128568. doi: 10.1001/jamanetworkopen.2021.28568 (PMC8515212; doi:10.1001/jamanetworkopen.2021.28568)
Supplement: Supplement. — eFigure 1. Flow Diagram for Systematic Review of PASC eFigure 2. PASC Frequencies Stratified by National Income Level, Proportion Hospitalized, and Study Methodological Quality [file jamanetwopen-e2128568-s001.pdf]

Supplemental Online Content

Groff D, Sun A, Ssentongo AE, et al. Short-term and long-term rates of postacute sequelae of SARS-CoV-2 infection: a systematic review. *JAMA Netw Open*. 2021;4(10):e2128568. doi:10.1001/jamanetworkopen.2021.28568

eFigure 1. Flow Diagram for Systematic Review of PASC

eFigure 2. PASC Frequencies Stratified by National Income Level, Proportion Hospitalized, and Study Methodological Quality

This supplemental material has been provided by the authors to give readers additional information about their work.

© 2021 Groff D et al. *JAMA Network Open.*

eFigure 1: Flow Diagram for Systematic Review of PASC

A total of 57 studies met full inclusion and were included in subsequent meta-analyses.

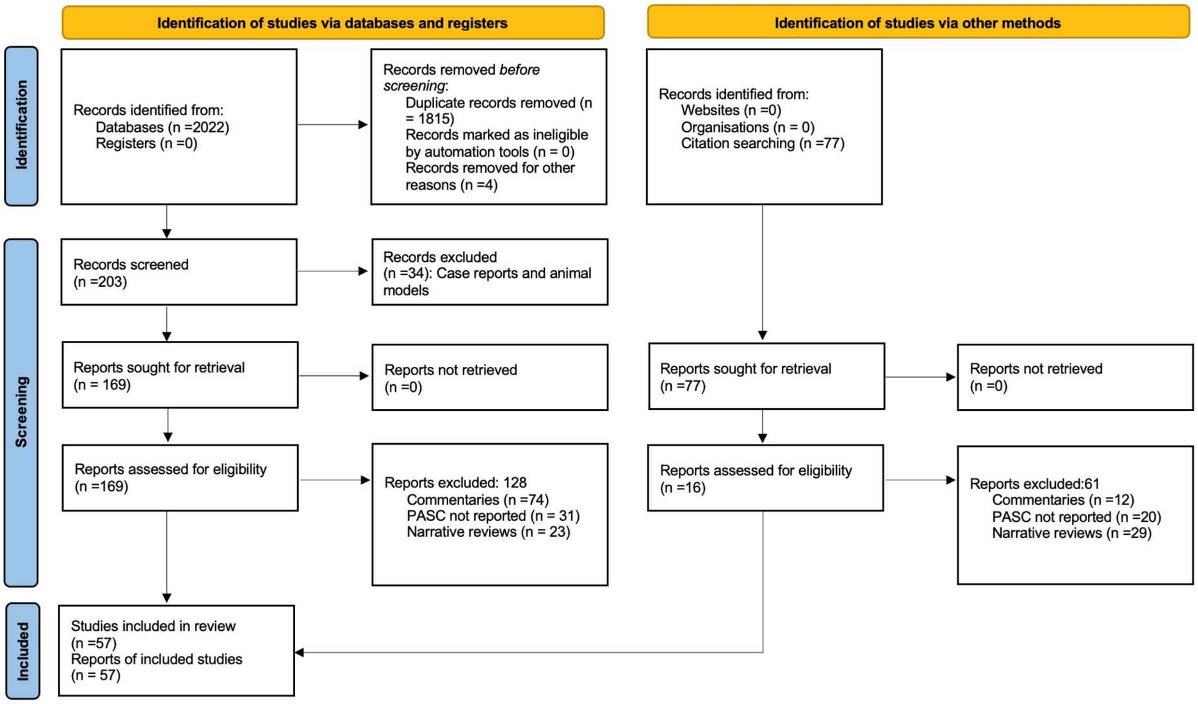

© 2021 Groff D et al. *JAMA Network Open.*

eFigure 2. PASC Frequencies Stratified by National Income Level, Proportion Hospitalized, and Study Methodological Quality

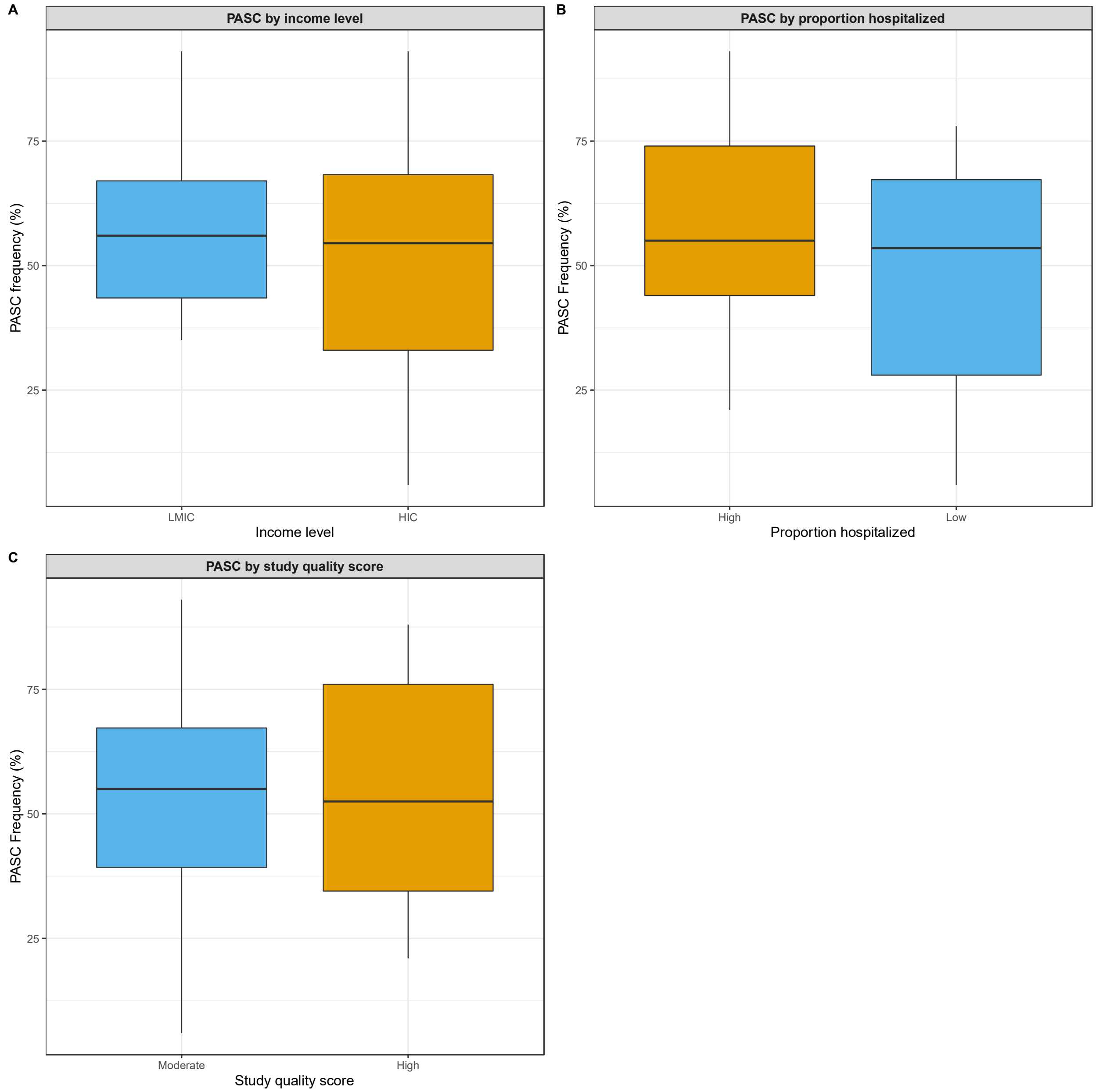

© 2021 Groff D et al. *JAMA Network Open.*
